# Combined effect of olive pruning residues and spent coffee grounds on *Pleurotus ostreatus* production, composition, and nutritional value

**DOI:** 10.1371/journal.pone.0255794

**Published:** 2021-09-24

**Authors:** Sami Abou Fayssal, Zeina El Sebaaly, Mohammed A. Alsanad, Rita Najjar, Michael Böhme, Milena H. Yordanova, Youssef N. Sassine

**Affiliations:** 1 Department of Agronomy, Faculty of Agronomy, University of Forestry, Sofia, Bulgaria; 2 Department of Plant Production, Faculty of Agriculture, Lebanese University, Beirut, Lebanon; 3 Department of Environment and Agricultural Natural Resources, College of Agricultural and Food Sciences, King Faisal University, Al Ahsa, Saudi Arabia; 4 Department of Physics, Faculty of Sciences IV, Lebanese University, Zahle, Lebanon; 5 Horticulture Department, Humboldt Universität zu Berlin, Berlin, Germany; University St. Kliment Ohridski - Bitola, THE FORMER YUGOSLAV REPUBLIC OF MACEDONIA

## Abstract

No previous study assessed the combined effect of olive pruning residues (OLPR) and spent coffee grounds (SCG) on *P*. *ostreatus* production and nutritional value. The aim of this study was to determine the capacity of *P*. *ostreatus* to degrade lignocellulosic nature of combined OLPR and SCG as well as their resultant nutrient composition. A complete randomized design was adopted with five treatments: S1:100%wheat straw (WS) (control), S2:33%WS+33%SCG+33%OLPR,S3:66%WS+17%SCG+17%OLPR,S4:17%WS+66%SCG+17%OLPR, and S5:17%WS+17%SCG+66%OLPR, and ten replicates per treatment. Substrate’s and mushroom’s composition were analyzed on chemical scale, including fatty acids and heavy metals profiles, following international standards. Only S1, S2, and S3 were productive, with comparable biological yield, economical yield, and biological efficiency. Organic matter loss decreased with increasing proportions of OLPR and SCG. Percentage lignin loss was higher in S1 than in S2 and S3 (53.51, 26.25, and 46.15% respectively). Mushrooms of S3 had some enhanced nutritional attributes compared to control: decrease in fat, increase in protein, increase in monounsaturated fatty acids, and lower zinc accumulation. Lead was less accumulated in S2 than S1 mushrooms. Sodium content of mushroom decreased in S2 and S3. The latter substrates yielded mushrooms with lower polyunsaturated fatty acids (PUFA) and higher saturated fatty acids (SFA) contents. All mushrooms had a valuable PUFA/SFA. This study suggests using OLPR and SCG in low proportions as nutritional supplements to the commercial wheat straw.

## Introduction

*Pleurotus* mushrooms can make an appreciable contribution to sustainable functional food design because of their special nutritional attributes [1]. *Pleurotus ostreatus* (Jacq.). P. Kumm. 1871 ranking second on the mushroom world production map [2] is especially appreciated due to its elevated protein content, high levels of thiamine, riboflavin, niacin, vitamin D, a valuable essential amino acid scoring pattern, high levels of essential minerals, like calcium, phosphorus, iron, zinc, and selenium, low sodium and fat contents, and a dominance of unsaturated fatty acids over saturated ones [1, 3–6]. *P*. *ostreatus* is also a low-calorific mushroom with important medicinal properties [7, 8].

*P*. *ostreatus* is a saprophyte, with high flexibility to grow by bioconversion on a wide range of lignocellulosic materials [9], like agricultural and industrial wastes [10]. In global commercial production, the mushroom is grown on wheat straw (WS) substrate [11], however, some constraints related to the high cost of straw may arise in a number of countries where wheat straw is scarce, such as in Lebanon. Successful implementation of agro-industrial wastes in mushroom production has been delineated in a number of studies [12–16]. Particularly, the study conducted by Abou Fayssal *et al*. [17] succeeded in reducing the dangerous hazard left over of olive pruning residues (OLPR) represented by open-field burning and fire risks. The production of a lower fat and sodium mushrooms with cardiovascular protection properties was also demonstrated. In the same vein, Alsanad *et al*. [18] found that recycling of spent coffee grounds (SCG) wastes by introducing them in oyster production is a method to reduce the negative impact of their disposal on the environment and to produce mushrooms of improved nutritional value.

The mushroom quality depends on the nutritional composition of the substrate used for its cultivation [19]. The mushroom capacity to absorb chemical compounds from the used substrate and the correlation between mushroom’s and substrate’s chemical composition has been demonstrated in a number of early reports [14, 20–23]. For instance, Abou Fayssal *et al*. [17] and Alsanad *et al*. [18] tested the potential of using abandoned OLPR or SCG on the production and nutritional value of *P*. *ostreatus* production, and demonstrated a direct impact of the substrate composition on the mushroom’s fatty acids profile. However, the combined use of both types of agro-industrial residues (OLPR and SCG) in *P*. *ostreatus* growth substrate is not reported yet. Therefore, in a continuous research chain to the latter studies, the present work addressed the combined effects of SCG and OLPR, incorporated in different proportions with wheat straw in the growing substrate, on the growth, production, and chemical composition of *P*. *ostreatus* mushroom.

## Materials and methods

### Experimental treatments

The study tested the effect of five different substrates containing OLPR and SCG mixed in different proportions of volume (0, 17, 33, or 66%) with WS. Tested substrates were: S1 (100%WS) (control), S2 (33%WS+33%SCG+33%OLPR), S3 (66%WS+17%SCG+17%OLPR), S4 (17%WS+66%SCG+17%OLPR), S5 (17%WS+17%SCG+66%OLPR). The experiment was arranged in a complete randomized design with five treatments (substrates) and ten replicates (10 bags per treatment).

### Spawning and incubation

Fresh wheat straw (sourced from a private farm), one-year fermented olive pruning residues (sourced from Compost Baladi, a local private company) and spent coffee grounds (sourced from Zero Waste Act, a waste segregation green project) were sun-dried for two days, then pasteurized using hot water (at 60–65°C) for eight hours and allowed to cool down to reach 25°C; spawning temperature. Olive pruning residues’ fermentation aims the degradation of lignocellulosic compounds in order to decrease the proportion of non-easily accessible holocelluloses and allow a higher energy for mycelial growth. After preparing the basic ingredients (WS, SCG, and OLPR), the five experimental mixtures were prepared by adding each ingredient according to the corresponding proportion of volume. Thus, five mixtures were ready for spawning, which was performed at 5% w/w rate, using wheat grain spawn of *P*. *ostreatus* M 2175 strain, (Mycelia Company, Deinze, Belgium). Spawned substrates filled into polyethylene bags (60 cm length × 40 cm width) were incubated in a cropping chamber with controlled environment following the criteria presented by Sassine et al. [24]. At the end of spawn run, colonized substrates were subjected to environmental triggers (lighting: 12 h day^-1^ of 200 LUX light source, reduction in room temperature to 16°C, reduction of air CO_2_ levels to < 900 mg L^-1^) to induce fruit formation.

### Evaluation of mushroom production

The stages of mycelia colonization (50% MC and 100% MC) were determined after observing and recording the time when half or all 5 × 5 cm squares drawn on bags were covered by mycelial patches, as described by Alsanad *et al*. [18]. The time to pinhead initiation (PN) and to first harvest (H1) were recorded as the number of days after spawning (DAS). Mushroom production was assessed by the number of harvests, number and weight of bunches (g bag^−1^), number and weight of mushrooms (g bag^−1^), biological yield (g bag^−1^), and economic yield (g bag^−1^). Stalk bases were removed before weighing the produced fruit bodies to assess the economical yield (g bag^−1^) for each treatment. Besides, the biological efficiency (BE) was calculated as the ratio of fresh mass of produced fruit bodies (G) over the initial substrate’s dry mass (g), and expressed as percentage. Organic matter loss (OML) corresponded to the ratio of the difference between initial and residual dry mass of the substrate (G) over the initial one, and expressed in percentage. Mushroom physical characteristics were assessed in terms of stipe diameter (SD) (cm), stipe length (SL) (cm) and pileus diameter (PD) (cm). The ratio PD/SL was also calculated on a sample of ten uniform mushrooms per treatment.

### Analytical tests

Various analytical tests were performed to determine the properties of initial substrates (Table 1), including their pH (by pH-meter: UltraBasic-UB10; Denver Instrument, New York, USA), electro-conductivity (by SC-2300 conductivity meter; Suntex Instrument, New Taipei City, Taiwan), organic matter content (by loss of ignition method at 430°C over 24 h), and C/N ratio (by CHN Analyzer with automatic sampler, Carlo-Erba elemental analyzer, Model 1106, Milan, Italy).

**Table 1:** Physicochemical characteristics of experimental substrates.

	S1	S2	S3	S4	S5
**pH**	5.5	7.7	6.7	5.4	6.2
**EC (ms/cm)**	0.3	1.84	0.72	1.27	2.41
**OM (%)**	86.6	82.1	84.7	93.5	83.3
**C/N ratio**	69:1	46:1	59:1	26:1	37:1

S1: 100%WS, S2: 33%WS+33%SCG+33%OLPR, S3: 66%WS+17%SCG+17%OLPR, S4: 17%WS+66%SCG+17%OLPR, S5: 17%WS+17%SCG+66%OLPR, WS: wheat straw, SCG: spent coffee grounds, OLPR: olive pruning residues, EC: electro–conductivity, OM: organic matter, C: carbon, N: nitrogen.

Moreover, the determination of the substrate’s total protein content followed the Association of Official Chemistry (AOAC) [25], using Micro-Kjeldahl method (N × 6.25). On mushrooms, the total protein content was estimated by the macro-Kjeldahl method (N × 4.38) [26]. The total carbohydrates content of both substrates and mushrooms were determined by the Anthrone method [27], and their fat content based on the protocols of the Association of Official Chemistry (AOAC) [25] using a Soxhlet apparatus for continuous extraction. Crude fiber content was determined using an enzymatic gravimetric method of analysis based on AOAC [28] protocol for substrates, and based on AOAC official general method 962.09 [29] for mushrooms. The analysis of sugar composition in substrates and mushrooms and fatty acid composition in mushrooms used High-Performance Liquid Chromatography (HPLC) and Gas Chromatography Mass Spectrophotometry (GC-MS), respectively, referring to the methods described in Alsanad *et al*. [18]. The substrate’s fatty acids were determined by gas chromatography-mass spectrometry, as proposed by Nieto and Chegwin [30]. Fiber fractions (cellulose, hemicellulose, lignin, neutral detergent fiber (NDF), acid detergent fiber (ADF), and acid detergent lignin (ADL)) were assessed on dry samples of initial and residual substrates by applying the ANKOM technology method filter bag technique [31–33]. The analysis of substrate’s mineral composition (K, Ca, Mg, Na, Fe, and Mn) applied the atomic absorption spectrophotometry method, based on AOAC [25]. For dry mushroom samples, mineral composition was determined using Inductively Coupled Plasma-Atomic Emission Spectrophotometry (ICP-AES) (ACTIVIA-M, Horiba Jobin Yvon) after element extraction in 0.1 N HCl acidic solutions. Phosphorus content was determined following the spectrophotometry method presented by AOAC [34] procedures. Zinc, copper, nickel, and lead were determined using an Atomic Absorption Spectrometer (AAS) (Analyst 700 Perkin Elmer, Massachusetts, USA) with air-acetylene burner for flame and ICP-AES with Argon plasma using AAS/ICP-AES instrument. A working standard based on commercially available multi-element standard solution (100 mg L^-1^, Merck, Germany) was used in order to calibrate the ICP-AES instrument. Practically, wave length, argon gas flow, plasma stabilization, and other ICP-AES instrument parameters for heavy metals were chosen to be the most appropriate following AOAC [35] calibrations. The different analytical tests (on substrates and mushrooms samples) were performed in pentuiplicates.

### Statistical analysis

One-way ANOVA and Duncan tests were applied for data analysis using SPSS 25® program. Stepwise regression between organic matter loss (OML) from the tested substrates and the proportions of SCG and OLPR was assessed. Moreover, Partial Least Squares Regression (PLSR) was applied for multivariate analysis, using XLSTAT. Jackknife (LOO) test was used for cross-validation of the resulting models. A confidence level of 95% was adopted for all statistical tests.

## Results and discussion

### Substrate effect on mushroom production

Substrates S4 and S5 weren’t fully colonized by the mycelium, they only attained a level of 50% mycelial colonization (MC). Early findings showed the possibility of gaining production from mixing high proportions of SCG or OLPR (67%) with low proportion of WS (33%) [17, 18]. However, in this study, when both tested wastes (SCG and OLPR) formed together 83% of substrate, the C/N ratio was strongly reduced (26:1 and 37:1 respectively in S4 and S5, compared to 69:1 in WS) (Table 1), assumedly causing a negative effect on mycelia run. According to Hoa *et al*. [36], the substrate C/N ratio is correlated with mycelial growth rate. Although the timing of spawn run initiation was similar in all substrates (Table 2), and in S4 and S5, the stage of 50% mycelia colonization occurred earlier by 1.3 and 2.0 days than control, it seems that the subsequent fungal growth was counteracted by the low C/N ratio of both substrates. In fact, carbon sources are obtained from the catabolic breakdown of the substrate organic compounds and are essential for the fungus to grow and perform its life activities [37].

**Table 2:** Substrate effect on various indicators of mushroom growth and production.

	S1	S2	S3	S4	S5	p-value	F
**SRI (DAS)**	1.0±0.0a	1.0±0.0a	1.0±0.0a	1.0±0.0a	1.0±0.0a	-	-
**50%MC (DAS)**	5.3±0.6c	4.7±0.6bc	5.0±0.0c	4.0±0.0ab	3.3±0.6a	0.002	9.67
**100%MC (DAS)**	7.7±1.1a	6.7±0.6a	7.7±0.6a	-	-	0.296	1.50
**PN (DAS)**	32.0±3.5a	30.7±0.6a	33.3±4.6a	-	-	0.643	0.47
**H1 (DAS)**	34.7±4.6a	32.7±0.6a	35.7±4.0a	-	-	0.602	0.55
**HN**	2.3±0.6a	4.0±0.0b	3.7±0.6b	-	-	0.011	10.50
**BN**	11.0±0.0b	4.0±1.0a	5.0±0.0a	-	-	<0.001	129.00
**BW (g)**	85.6±28.0a	210.0±52.8b	176.7±13.3b	-	-	0.012	9.94
**FBN**	32.7±2.5a	47.0±7.0b	52.7±7.5b	-	-	0.018	8.54
**FBW (g)**	13.1±2.8a	17.0±2.1a	16.5±2.1a	-	-	0.169	2.43
**BY (g)**	910.1±236.3a	811.7±13.7a	883.6±66.5a	-	-	0.696	0.39
**EY (g)**	871.4±235.0a	782.4±22.7a	861.1±66.7a	-	-	0.715	0.35
**BE (%)**	105.0±27.2a	95.3±2.7a	101.7±7.9a	-	-	0.771	0.27

S1: 100%WS, S2: 33%WS+33%SCG+33%OLPR, S3: 66%WS+17%SCG+17%OLPR, S4: 17%WS+66%SCG+17%OLPR, S5: 17%WS+17%SCG+66%OLPR, SRI: spawn run initiation, MC: mycelial colonization, PN: time to pin head initiation, H1: time to first harvest, HN: harvests number, BN: bunches number, BW: bunches weight, FBN: fruit body number, FBW: fruit body weight, BY: biological yield, BE: biological efficiency, EY: economic yield, DAS: days after spawning. Values are means ± SD; means within the same row followed by the same letters are not significantly different at *p* < 0.05 according to Duncan’s multiple range test.

Further, when wheat straw was present in equal proportions to SCG and OLPR (S2) or dominant over both wastes (S3), mycelia run was unaltered; timings of 100% MC, pin head initiation, and first harvest were comparable to control (very low statistical F). But, the presence of SCG and OLPR in the substrate has exhibited a more pronounced effect at the productive stage; significant increase in harvest number (by 1.7 and 1.4), bunch weight (by 124.4 g and 91.1 g), and fruit body number (by 14.3 and 20.0), and a significant decrease in bunches number (by 7.0 and 6.0) in S2 and S3, respectively compared to control. On the other hand, fruit body weight remained unchanged; comparable values in S2, S3, and control.

Biological yield, economical yield, and biological efficiency (BE) were comparable between all productive substrates with a slight (but non-statistically significant) superiority of the three indicators in control compared to S2 (by 98.4 g, 89 g, and 9.7%, respectively) and S3 (by 26.5 g, 10.3 g, and 3.3%, respectively). The combined yield of mushrooms harvested from two flushes of *P*. *ostreatus* cultivated in grass and coffee pulp produced a BE varying between 59.9 and 93% [38].

**Fig 1:**
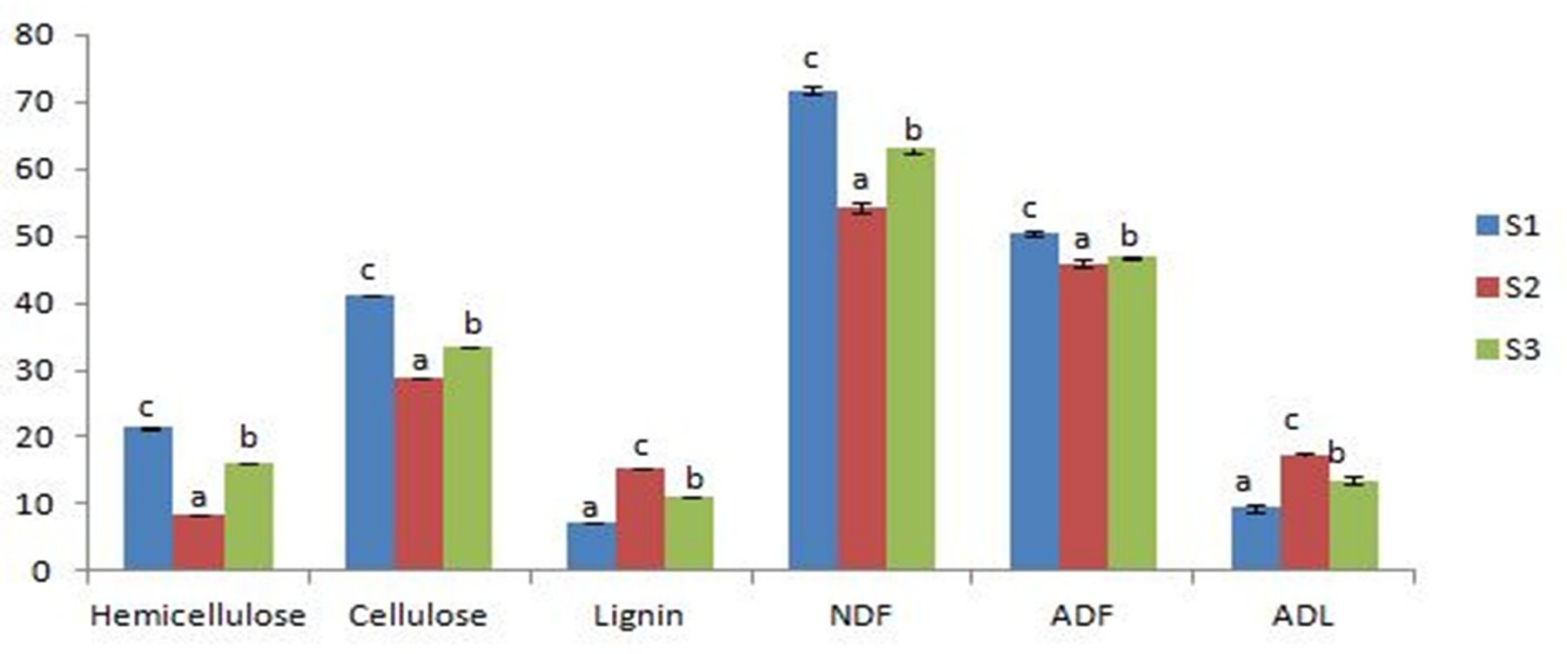
Fiber fractions analysis (% dry weight) of initial tested substrates. Values are means ± SD; for each indicator, different letters indicate a statistically significant difference at *p* < 0.05; S1:100%WS, S2:33%WS+33%SCG+33%OLPR, S3: 66%WS+17%SCG+17%OLPR.

The slight decrease in the biological yield in the substrates S2 and S3 could associate to their higher lignin and lower cellulose and hemicellulose contents compared to S1 (Fig 1). White rot fungus (*P*. *ostreatus*) can degrade the substrate’s lignocellulosic complex into soluble sugars, essential for its growth through the action of complex oxidative and hydrolytic enzymatic systems [39, 40]. For instance, upon break, cellulose yields simple sugars, which provide energy for the mycelial growth [41]. To access the substrate holocellulose (cellulose and hemicellulose), the fungus needs first to degrade lignin [42]. A higher lignin loss means higher mycelial activity [43].

**Fig 2:**
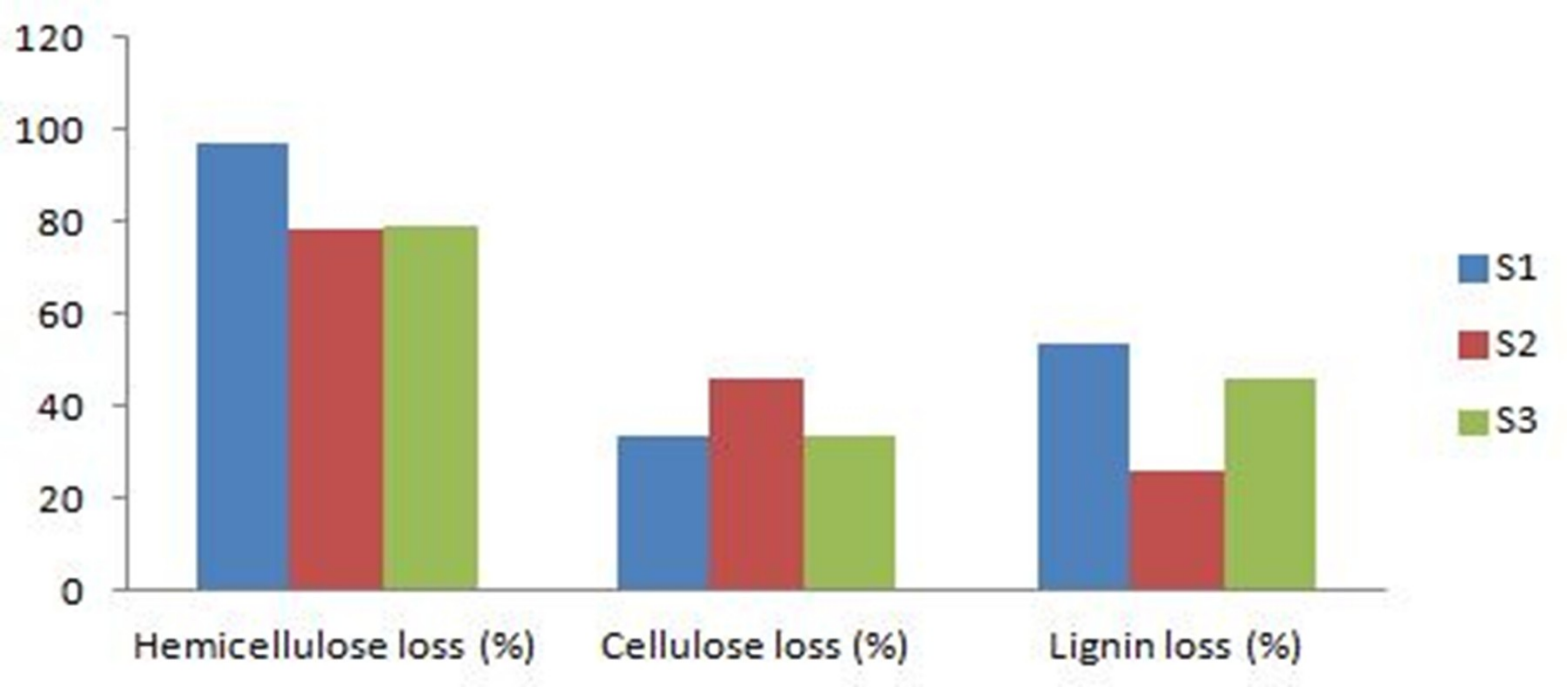
Percentage loss of hemicellulose, cellulose and lignin from tested substrates. S1:100%WS, S2:33%WS+33%SCG+33%OLPR, S3:66%WS+17%SCG+17%OLPR.

Percentage loss of lignin was higher in S1 than in S2 and S3 (53.51, 26.25, and 46.15% respectively) (Fig 2), reflecting a better mycelia growth and causing the slightly higher production in S1. Further, in all tested substrates, hemicellulose was preferentially degraded with respect to cellulose, which is consistent to early findings of Thompson *et al*. [44] obtained on wheat straw.

**Fig 3:**
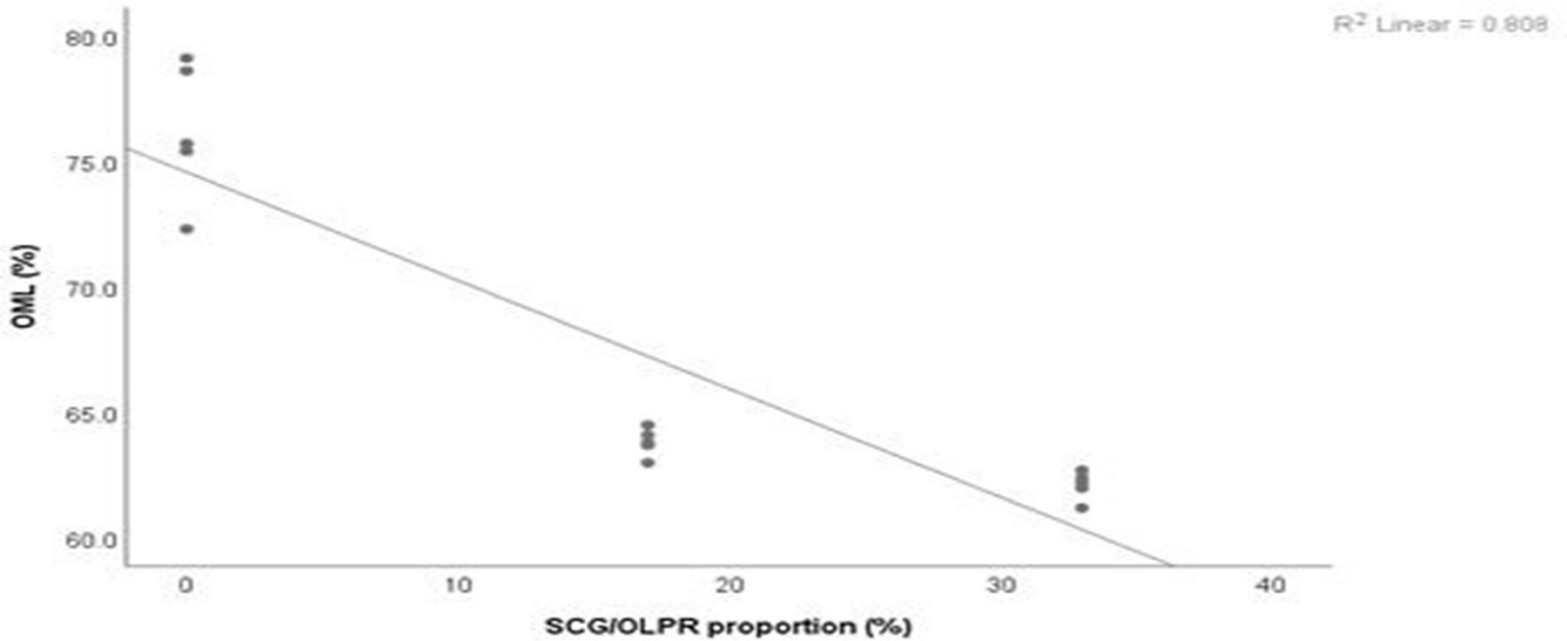
Relation between OML and proportions of SCG and OLPR (SCG/OLPR). OML = −0.43 × SCG/OLPR + 74.56; *r*^2^ = 0.81.

Moreover, results of the regression analysis (Fig 3) delineated a strong linear relationship (*r*^2^ = 0.81) between the proportions of SCG and OLPR and organic matter loss (OML) from the tested substrates. In particular, there was a gradual decrease in OML, thus in the degradation rate, of substrates containing increasing proportions of SCG and OLPR (average values of OML were respectively of 76.2, 63.8, and 62.1% in S1, S3, and S2). This relationship might explain the slight gradual decrease in the biological efficiency in such substrates, compared to the wheat straw substrate. Isikhuemhen *et al*. [45] found a positive correlation between *P*. *ostreatus* biological efficiency and OML obtained in different mixtures of solid waste, wheat straw, and millet.

### Substrate effect on mushroom appearance

**Fig 4:**
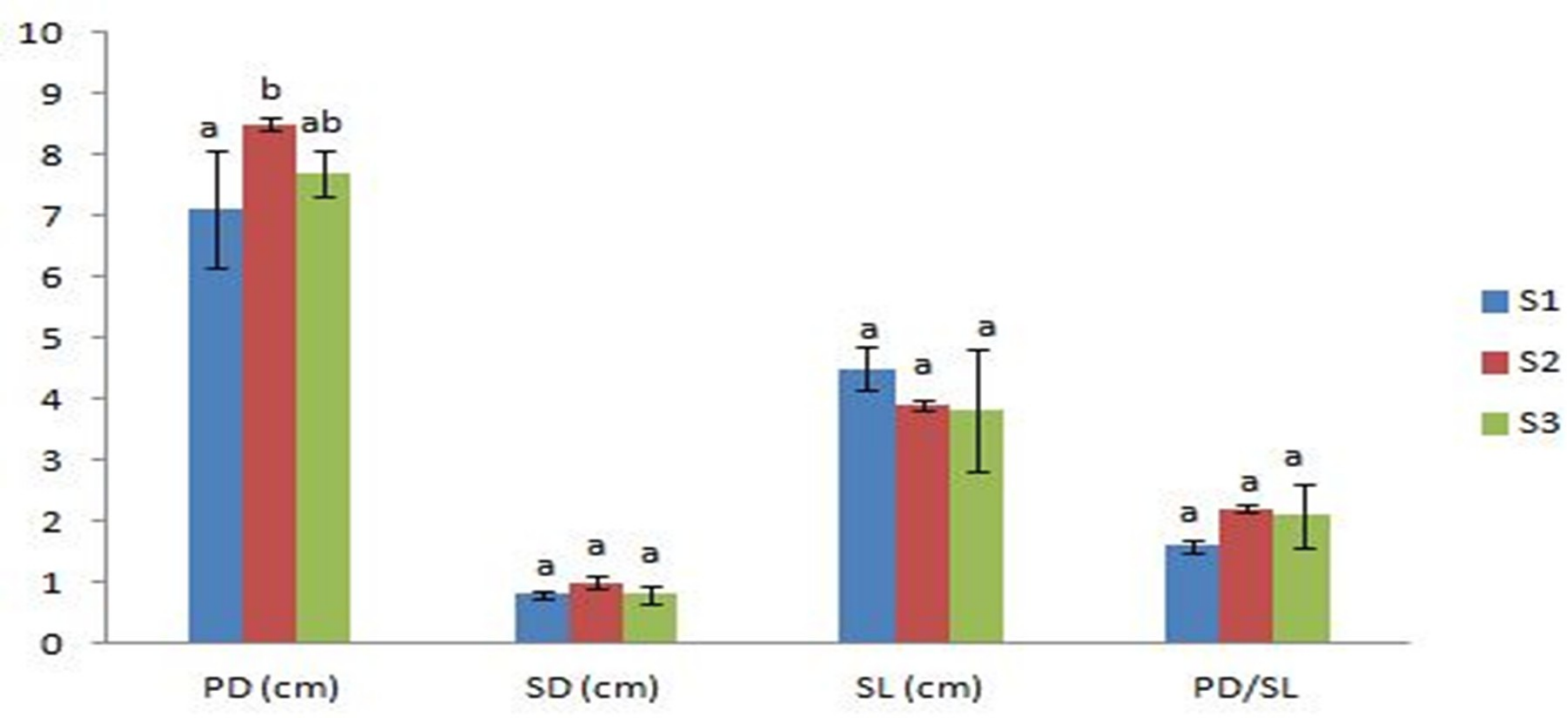
Pileus and stipe characteristics of mushrooms grown on different substrates. Values are means ± SD; for each indicator, means followed by the same letter are not statistically significant at *p* < 0.05 according to Duncan’s multiple range test; S1: 100%WS, S2: 33%WS+33%SCG+33%OLPR, S3: 66%WS+17%SCG+17%OLPR, SD: stipe diameter, PD: pileus diameter, SL: stipe length, WS: wheat straw, SCG: spent coffee grounds, OLPR: olive pruning residues.

The substrate type didn’t have a statistically significant effect on the average of pileus diameter (*p* = 0.074), stipe diameter (*p* = 0.174), stipe length (*p* = 0.403), and PD/SL (*p* = 0.122) (Fig 4). Nevertheless, the pileus diameter of mushrooms produced in S2 increased by 1.4 cm in comparison with control mushrooms. Mushrooms obtained in all treatments were marketable showing a high PD/SL ratio [46] with promising values of PD/SL for mushrooms harvested from substrates containing SCG and OLPR.

### Effect of substrate on mushroom composition

Carbohydrates were lower in mushrooms of S2 and S3, although both substrates contained comparable or higher carbohydrates content compared to S1 (Table 3). Mushrooms’ carbohydrates are mostly non-digestible ones, including oligosaccharides such as trehalose and cell wall polysaccharides, such as chitin, β-glucans, and mannans [47], therefore, the presence of SCG and OLPR in the substrate may have indirectly influenced the mushroom firmness and texture. In a similar pattern, mushrooms’ crude fiber content decreased in S2 and S3 compared to control. The crude fiber of mushrooms, as a type of dietary fiber, contains mainly the water-insoluble fiber fraction, with chitin and β-glucans being the most representative ones. Mushroom’s dietary fibers present many beneficial health benefits, including immune-enhancing, antitumor activity, novel prebiotics, and blood glucose and lipid attenuation [48, 49]. On the nutritional level, a low-carbohydrate diet could be favorable for improving the body composition and high-density lipoprotein (HDL) cholesterol level [50], but a low-fiber diet could be only beneficial for a specific therapy of gastrointestinal problems [51]. Control substrate was the richest in total soluble sugars including fructose, glucose, and sucrose contents in comparison with S2 and S3. Richness in soluble sugars is directly linked to the strongest growth and highest production obtained in control substrate, because mushrooms utilize these soluble sugars as a source of energy to colonize the substrate [39]. On the other hand, control mushrooms were the poorest in total soluble sugars and fructose contents and the richest in glucose content in comparison with mushrooms obtained in S2 and S3. In particular, sugar content of mushrooms increased as long as OLPR and SCG proportions increased in the substrates, to be almost double in mushrooms of S2 compared to S1. Such gradual increase in mushroom’s total soluble solids was similarly observed by Abou Fayssal *et al*. [17] and Alsanad *et al*. [18] after adding OLPR and SCG, each apart, and in increasing proportions to wheat straw. Moreover, S2 and S3 were richer in total protein content than control substrate (by 5.40 and 0.30%, respectively). Protein content was superior in S3 mushrooms, where it was higher by 0.15% than control mushrooms. Protein is an important constituent of mushrooms dry matter; because of their high protein content, mushrooms rank between meat and vegetables [52]. Also, Phillips *et al*. [53] suggested consuming protein foods as they contribute in better diet quality and nutrient adequacy. Fat content was higher in substrates S2 and S3 compared to S1, while in mushrooms; it increased by 0.14% in S2 and decreased by 0.2% in S3 compared to control mushrooms. Early findings of Abou Fayssal *et al*. [17] proved that mixing OLPR in low or high proportions with wheat straw reduced the mushroom’s fat content. Therefore, superior fat content in S2 could be most probably caused by the SCG presence in this substrate. *Pleurotus* spp. is low in fat. Overall, the values of mushroom fat content obtained in all treatments of this study were lower than early reports [1].

**Table 3:** Mushrooms (% fresh weight) and initial substrates (% dry weight) composition.

	Carb	CF	TSS	FRU	GLC	SUC	Pro	Fat
**Substrates**								
**S1**	38.54±0.05a	38.44±0.01a	1.54±0.05b	0.60±0.01b	0.74±0.01c	0.20b	5.50±0.008a	0.73±0.02a
**S2**	38.53±0.02a	38.43±0.02a	0.16±0.02a	0.02±0.002a	0.14±0.02b	<0.005a	10.90±0.02c	1.46±0.02b
**S3**	39.16±0.02b	39.06±0.02b	0.12±0.02a	0.02±0.002a	0.10±0.02a	<0.005a	5.80±0.02b	1.52±0.02c
**p-value**	<0.001	<0.001	<0.001	<0.001	<0.001	<0.001	<0.001	<0.001
**F**	701.56	2641.84	3226.15	8325.86	2739.05	247.19	245939.65	3696.43
**Mushrooms**								
**S1**	6.19±0.004C	3.90±0.003C	0.013±0.002A	0.007±0.002A	0.007±0.002B	<0.005	2.94±0.003B	0.19±0.002B
**S2**	4.69±0.002B	2.51±0.002B	0.026±0.002C	0.024±0.002C	0.002±0.0002A	<0.005	2.16±0.002A	0.33±0.002C
**S3**	4.02±0.002A	2.25±0.04A	0.017±0.002B	0.01±0.002B	0.007±0.0002B	<0.005	3.09±0.002C	0.17±0.002A
**p-value**	<0.001	<0.001	<0.001	<0.001	<0.001	-	<0.001	<0.001
**F**	894839.14	7171.80	67.93	164.67	31.22	-	271069.62	12232.34

S1: 100%WS, S2: 33%WS+33%SCG+33%OLPR, S3: 66%WS+17%SCG+17%OLPR, Carb: total carbohydrates, CF: crude fiber, TSS: total soluble sugars, FRU: fructose, GLC: glucose, SUC: sucrose, Pro: total protein. Values are means ± SD; means within the same column followed by the same letters of lower case–corresponding to substrates–or uppercase–corresponding to mushrooms–are not significantly different at *p* < 0.05 according to Duncan’s multiple range test.

According to Kalač and Svoboda [54], the mushroom’s mineral levels are greatly affected by the growth substrates. In other terms, substrates high in a particular mineral yield mushrooms relatively high in the content of that mineral. However, this was not always true in this study, as mineral contents were in some cases high in substrates, but low in mushrooms, or vice-versa. In fact, the analysis of mineral composition (Table 4) revealed higher iron and magnesium contents in substrates containing SCG and OLPR in comparison with control. However, only magnesium content has significantly increased in mushrooms produced by S2 and S3, compared to control mushrooms (by 0.006 and 0.004% respectively). Calcium content was higher in S2 by 0.34% in comparison with control, but mushrooms produced by both substrates had comparable calcium contents. Moreover, potassium and manganese contents in S1 substrate were higher by 0.41 and 0.07% for the former and 0.0075 and 0.0078% for the latter compared with S2 and S3, respectively. On the contrary, mushrooms of S2 and S3 were richer by 0.07% in potassium and by 0.00002 and 0.0008% respectively in manganese, compared with S1 mushrooms. Sodium content was significantly lower in S2 and S3 substrates and mushrooms compared to control cases. In general, the increase in potassium coupled with the reduction in sodium content in mushrooms, caused by the combined presence of SCG and OLPR in the substrate, is beneficial from a nutritional point of view. Such distinctive nutritional aspect allows reducing blood pressure and the risk of heart diseases [1]. A reduced sodium intake could also help fighting heart failure and chronic kidney disease [55].

**Table 4:** Mineral composition of initial substrates and mushrooms (% dry weight).

	Fe	Mg	Ca	K	Mn	Na
**Substrates**						
**S1**	0.015±0.002a	0.08±0.003a	0.42±0.004a	1.43±0.01c	0.08±0.003b	0.07±0.004b
**S2**	0.16±0.02c	0.18±0.002c	0.76±0.002b	1.02±0.02a	0.005±0.0002a	0.06±0.000a
**S3**	0.07±0.002b	0.12±0.002b	0.41±0.002a	1.36±0.02b	0.002±0.0002a	0.06±0.000a
**p-value**	<0.001	<0.001	<0.001	<0.001	<0.001	<0.001
**F**	307.93	140.33	24669.57	1097.08	4182.52	32.74
**Mushrooms**						
**S1**	0.002±0.000B	0.013±0.002A	0.0025±0.000B	0.25±0.002A	0.00010±0.000A	0.042±0.002B
**S2**	0.002±0.000B	0.019±0.000C	0.0027±0.000B	0.32±0.000B	0.00012±0.000B	0.007±0.000A
**S3**	0.001±0.000A	0.017±0.000B	0.0015±0.000A	0.32±0.000B	0.00090±0.000C	0.007±0.000A
**p-value**	0.060	<0.001	<0.001	<0.001	<0.001	<0.001
**F**	3.60	39.02	27.81	5921.37	69767.10	2435.96

S1: 100%WS, S2: 33%WS+33%SCG+33%OLPR, S3: 66%WS+17%SCG+17%OLPR. Values are means ± SD; means within the same column followed by the same letters of lower case–corresponding to substrates–or uppercase–corresponding to mushrooms–are not significantly different at *p* < 0.05 according to Duncan’s multiple range test.

### Fatty acid profile of substrates and mushrooms

Before moving into the analysis of fatty acids composition of the different mixtures, it is essential to provide the initial composition of pure SCG and OLPR. The fatty acid profile of pure SCG was as follows: 41.2% linoleic acid, 34.91% palmitic acid, 9.7% oleic acid, 9.6% stearic acid, 3.6% linolenic acid, and 0.9% arachidic acid. Pure OLPR contained 66.96% linoleic acid, 11.08% oleic acid, 10.64% palmitic acid, 6.17% linolenic acid, 3.47% stearic acid, and 1.67% arachidic acid.

Results in Table 5 showed that linoleic acid was more abundant in S2 and S3 substrates compared to S1, due to the richness of pure SCG and OLPR in this fatty acid. Linoleic acid was the most abundant fatty acids in mushrooms produced by all tested substrates, and it was superior in mushrooms obtained in S1. Linolenic acid content lower in substrates containing SCG+OLPR compared to control. Initially, pure SCG and OLPR were poorer in this fatty acid compared to wheat straw. However, in S3 mushrooms, the content of this fatty acid was significantly higher than in mushrooms of control substrate. The palmitoleic acid found in wheat straw, was absent in substrates containing SCG+OLPR. This fatty acid was initially absent in pure SCG and OLPR. However, it was found in mushrooms produced in S2 and S3, which suggests wheat straw as a source of this fatty acid. Previously, Alsanad *et al*. [18] mentioned that the palmitoleic may be originated from wheat straw. Oleic acid, initially more abundant in S2 and S3 substrates, was significantly lower in mushrooms of the former (reduction by 3.04%) and higher in those of the latter (increase by 8.21%), compared to control mushrooms. Myristic acid absent in all productive substrates was found in mushrooms produced by S2 and S3. As this fatty acid was initially absent in pure SCG and OLPR, it may be indirectly synthesized in the mushroom, which proves the conclusive findings of Alsanad *et al*. [18]. Moreover, the palmitic acid was initially more abundant in S1, but its content in mushrooms increased significantly in S2 and S3 to be almost double in the latter compared to S1. The substrates S2 and S3 as well as their mushrooms were richer in terms of stearic acid in comparison with control substrate and mushrooms. This could be due to the combined presence of OLPR and SCG, initially rich in stearic acid, with WS containing good amounts of this fatty acid. Arachidic acid, initially present in pure SCG and OLPR, was detected in S2 and S3, but not in S1. Its absence in mushrooms produced by all tested substrates may suggest its complete conversion at the level of the mushroom into other components, confirming the findings of Abou Fayssal *et al*. [17] and Alsanad *et al*. [18].

**Table 5:** Fatty acids profiles of initial substrates and mushrooms (% dry weight).

	PUFA		MUFA		SFA			
Substrates	C18:2	C18:3	C16:1	C18:1	C14:0	C16:0	C18:0	C20:0
**S1**	21.06±0.003a	8.06±0.002c	1.20±0.004a	10.48±0.002a	nd	53.32±0.003c	5.88±0.007a	Nd
**S2**	40.42±0.000b	3.34±0.000a	Nd	12.10±0.000c	nd	34.15±0.000a	8.37±0.000b	1.60±0.000a
**S3**	40.59±0.000c	3.35±0.000b	Nd	10.62±0.000b	nd	35.31±0.000b	8.44±0.000c	1.69±0.000b
**p-value**	<0.001	<0.001	-	<0.001	-	<0.001	<0.001	<0.001
**F**	154772778.80	25755925.93	-	3666300.55	-	153967765.80	636138.32	19781356.50
**Mushrooms**								
**S1**	80.59±0.009C	0.20±0.002A	0.09±0.003A	9.10±0.002B	nd	9.18±0.01A	0.84±0.003A	Nd
**S2**	65.98±0.000B	0.21±0.000A	0.45±0.003C	6.06±0.000A	0.35±0.002A	14.05±0.000B	0.90±0.000B	Nd
**S3**	60.02±0.000A	0.33±0.000B	0.39±0.000B	17.31±0.002C	0.33±0.002A	18.71±0.000C	2.91±0.000C	Nd
**p-value**	<0.001	<0.001	<0.001	<0.001	<0.001	<0.001	<0.001	-
**F**	21040017.21	1408969.00	36395121.79	61793987.46	1579.15	1644603.15	2512149.55	-

S1: 100%WS, S2: 33%WS+33%SCG+33%OLPR, S3: 66%WS+17%SCG+17%OLPR. PUFA: polyunsaturated fatty acids, MUFA: monounsaturated fatty acids, SFA: saturated fatty acids, C18:2: linoleic acid, C18:3: linolenic acid, C16:1: palmitoleic acid, C18:1: oleic acid, C14:0 myristic acid, C16:0: palmitic acid, C18:0: stearic acid, C20:0: arachidic acid, nd: not detected. Values are means ± SD; means within the same column followed by the same letters of lower case–corresponding to substrates–or uppercase–corresponding to mushrooms–are not significantly different at *p* < 0.05 according to Duncan’s multiple range test.

Polyunsaturated fatty acids were the highest in control mushrooms (around 80.8% compared to 66.2% and 60.3% in S2 and S3, respectively). Monounsaturated fatty acids (MUFA) decreased in S2 mushrooms in comparison with control ones (by around 2.7%) and increased in S3 mushrooms (by 8.5%). In terms of human dietary intakes, monounsaturated fatty acids help reducing the low-density-lipoprotein (LDL) [56]. Saturated fatty acids were higher in mushrooms of S2 and S3 in comparison with those of control (increase by around 4.9% and 11.6%, respectively). This type of fatty acids increase LDL levels and lead to diabetes [57]. The ratio PUFA/SFA was the highest in control mushrooms (8.1), followed by S2 (4.4) and S3 mushrooms (2.8). It was reported that foods with relatively high PUFA/SFA are healthy and present a strong hypocholesterolemic effect [58]. In their study on wild strains of *P*. *ostreatus*, Koutrotsios *et al*. [59] reported that PUFA were the most dominant, ranging between 58.84% and 80.63%, while MUFA ranged between 6.76% and 20.29% and SFA were between 8.77 and 17.07% of total fatty acids. They also found linoleic acid as the most prevalent in mushrooms, followed by oleic and palmitic acids. In the present study, palmitic acid was more abundant than oleic acid in all treatments.

### Correlation between mushroom fatty acids and substrate composition

The interrelationships between the initial substrates’ components, including minerals, fatty acids, total protein, carbohydrates, fat, crude fiber, and others (as independent variables represented by the X-matrix) and the mushroom fatty acids (as dependent variables represented by the Y-matrix) were defined by the following partial least squares regression (PLSR) analysis. The difference between observed and predicted values of a model was defined by the root means square error (RMSE). The latter was low for all built models (ranging between 0.001 and 0.331) (Table 6) indicating a well fit model for the present experimental data. Q^2^cum is the cross-validation tool that states the stability of the model. It also sets the lower bound of how well the model explains the data. R^2^Ycum is the coefficient between Y and (t1, t2). It gives an upper bound of how well the model explains the data and predicts new observations.

**Table 6:** Overview of PLSR model.

		Q^2^cum		R^2^Ycum	
Mushroom fatty acid	RMSE	Comp 1	Comp 2	Comp 1	Comp 2
**C14:0 myristic acid**	0.001	0.422	0.986	0.570	0.997
**C16:0 palmitic acid**	0.153	0.657	0.995		
**C16:1 palmitoleic acid**	0.006	0.980	0.998		
**C18:0 stearic acid**	0.057	0.111	0.991		
**C18:1 oleic acid**	0.331	0.089	0.989		
**C18:2 linoleic acid**	0.300	0.856	0.997		
**C18:3 linolenic acid**	0.003	0.139	0.990		

RMSE: root mean square error, Q^2^cum: cross–validation tool, R^2^Ycum: coefficient between Y and (t1, t2), Comp: component.

**Fig 5:**
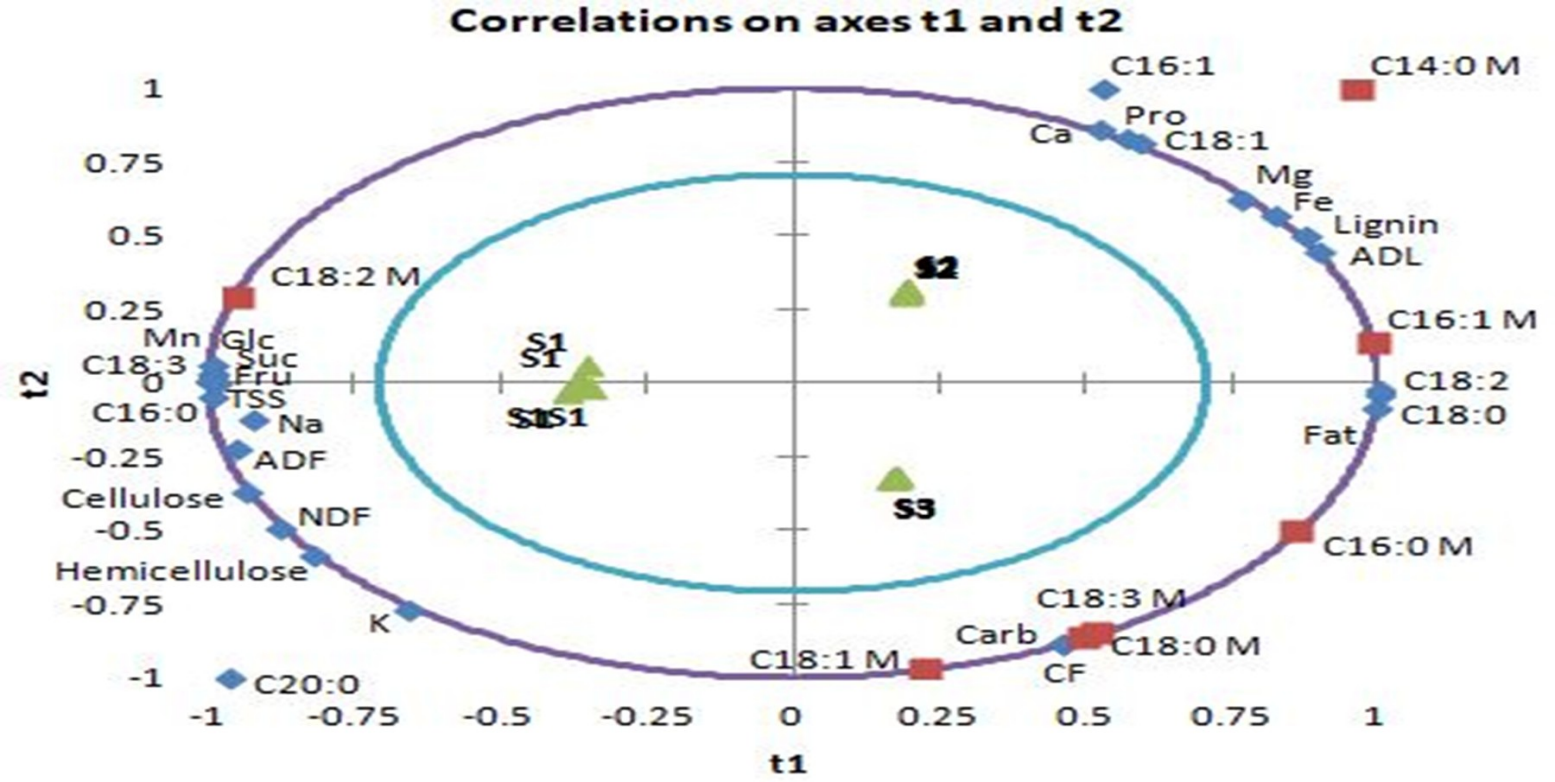
Correlations between mushroom fatty acids and substrates chemical composition. S1: 100%WS, S2: 33%WS+33%SCG+33%OLPR, S3: 66%WS+17%SCG+17%OLPR, C14:0: myristic acid, C16:0: palmitic acid, C16:1: palmitoleic acid, C18:0: stearic acid, C18:1: oleic acid, C18:2: linoleic acid, C18:3: linolenic acid, C20:0: arachidic acid, NDF: neutral detergent fiber, ADF: acid detergent fiber, ADL: acid detergent lignin, M: mushroom.

Correlations on axes t1 and t2 between palmitic, palmitoleic, stearic, oleic, linoleic, and linolenic acids, and the chemical components of tested substrates showed two components for X variables and two components for Y variables. In Fig 5, the presence of X variables (except: arachidic acid and palmitoleic acid contents of substrates) and Y variables (except: myristic acid content of mushrooms) between the inner and outer ellipses, indicates that correlations between these variables can be well explained by the relative PLSR model.

**Fig 6:**
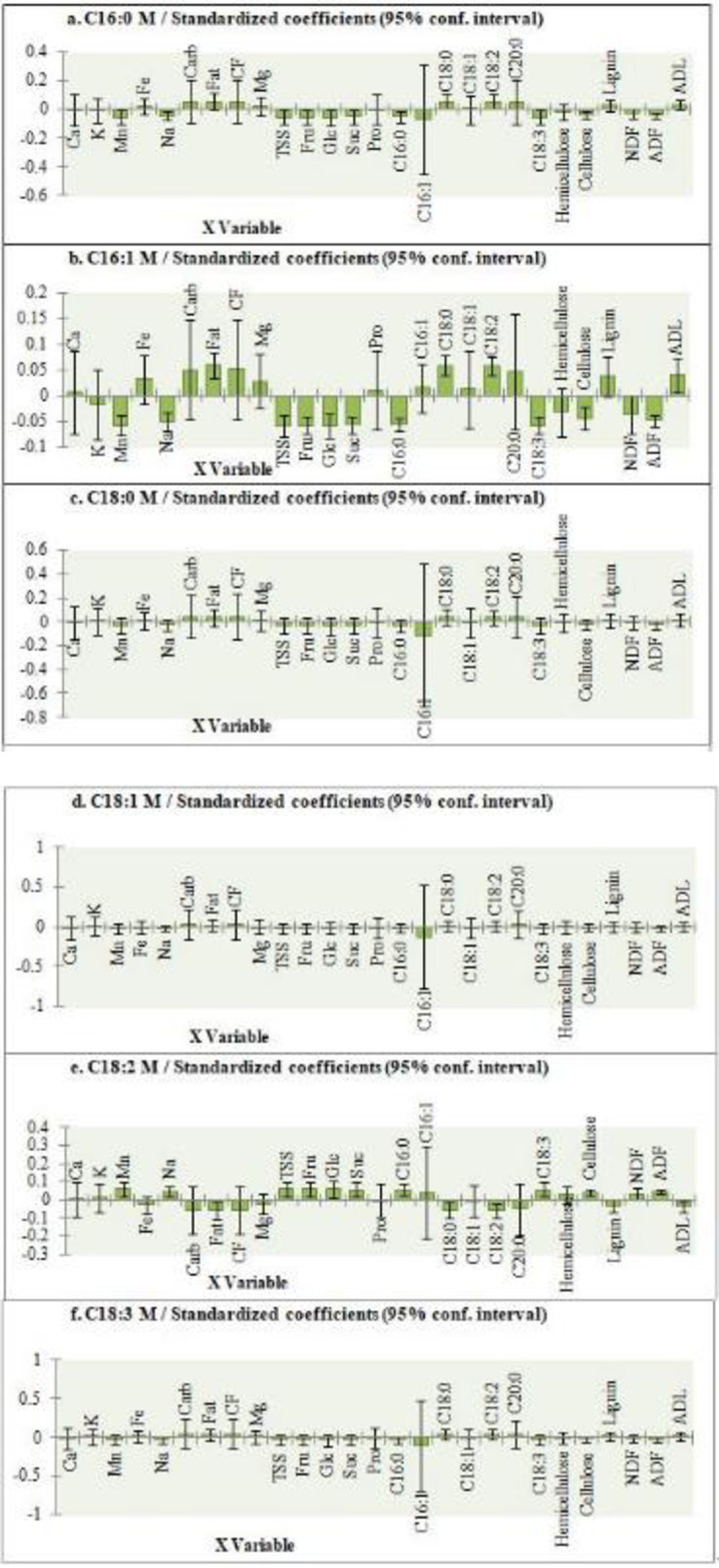
**Regression coefficients and significant indications (shown in streaked bars) for substrate chemical composition variable.** C14:0: myristic acid, C16:0: palmitic acid, C16:1: palmitoleic acid, C18:0: stearic acid, C18:1: oleic acid, C18:2: linoleic acid, C18:3: linolenic acid, C20:0: arachidic acid, NDF: neutral detergent fiber, ADF: acid detergent fiber, ADL: acid detergent lignin, Carb: carbohydrates, CF: crude fibers, Pro: proteins, TSS: total soluble solids, Fru: fructose, Glu: glucose, Suc: sucrose.

Furthermore, regression coefficients (Fig 6A–6F) were calculated in order to determine significant contribution of X variables to variation of relative Y variables. The predictive model (Fig 5) and the regression coefficients (Fig 6A) proved a co-variation and a positive correlation of the mushroom palmitic acid with stearic and linoleic acids present in S3 substrate. Palmitoleic acid of mushrooms co-varied (Fig 5) and was positively correlated (Fig 6B) with fat, stearic and linoleic acids of S3 substrate, and with lignin and ADL contents of S2 substrate. This mushroom fatty acid was negatively correlated with soluble sugars (including fructose, glucose and sucrose), manganese, and linolenic acid contents of S1 substrate. The strong correlation between palmitoleic acid content in mushrooms and lignin and ADL contents of S2 substrate suggests lignin as one of the main sources of this fatty acid. Stearic, oleic, and linolenic acids in mushrooms showed a co-variation (Fig 5) and were the most positively correlated (Fig 6C, 6D and 6F) with total carbohydrates and crude fiber contents found in S3 substrate, which leads to the assumption that these fatty acids were mainly originated from the main source of energy present in the substrate. In fact, the substrate S3 was the richest in carbohydrates and crude fibers and yielded mushrooms of superior contents of the three fatty acids compared to other mushrooms. Mushroom linoleic acid co-varied (Fig 5) and was positively correlated (Fig 6E) with total soluble sugars including fructose, glucose and sucrose, manganese, and linolenic acid contents of S1 substrate. This fatty acid found abundantly in mushrooms was negatively correlated with fat, stearic, and linoleic acids contents of S3 substrate. Linoleic acid present in substrate could have been converted into other types of fatty acids rather than being converted directly to the same type of fatty acid in mushrooms.

### Heavy metals profile in mushrooms

Despite their short-life span, mushrooms were reported to have a specific mechanism to effectively absorb heavy metals from the environment [60]. Oyster mushroom like other mushrooms can absorb heavy metals from substrates via spacious mycelium [61]. Several fungal factors, like the mushroom species, biochemical composition, morphological structure of the fruiting body, age of mycelium, and development stage can influence the mushroom content in heavy metals [54]. Besides, environmental factors like the amount of heavy metals in the substrates can primarily affect heavy metals contents in mushrooms [62]. Phosphorus content was slightly increased in mushrooms produced by S2 and S3 in comparison with control ones (increase by 0.08 and 0.1% respectively) (Table 7).

**Table 7:** Mushrooms composition (% dry weight) in heavy metals.

	P (%)	Cu (ppm)	Zn (ppm)	Ni (ppm)	Pb (ppm)
**S1**	0.61±0.02A	4.70±0.2A	72.00±0.2B	10.50±0.2A	23.00±0.2B
**S2**	0.69±0.02B	14.20±0.2C	72.80±0.2C	15.70±0.2C	22.70±0.2A
**S3**	0.71±0.02B	8.70±0.2B	59.30±0.2A	13.60±0.2B	25.40±0.2C
**p-value**	<0.001	<0.001	<0.001	<0.001	<0.001
**F**	56.00	4550.00	11472.67	1368.67	438.00

S1: 100%WS, S2: 33%WS+33%SCG+33%OLPR, S3: 66%WS+17%SCG+17%OLPR. ppm: parts per million. Values are means ± SD; means within the same column followed by the same letters are not significantly different at *p* < 0.05 according to Duncan’s multiple range test.

Çağlarırmak [63] reported that *P*. *ostreatus* is a phosphorus rich mushroom which makes it a good contributor in human nutrition. However, high levels of phosphorus inhibit the intake of calcium causing bones weakness, itchy skin and bone or joints pain leading to chronic kidney disease-mineral bone disorder [64]. Copper content in S2 and S3 mushrooms was higher by 9.5 and 4% respectively compared to its content in control mushrooms, but they were lower than that reported by Gebrelibanos *et al*. [65] (51.19 ppm) and in accordance with the FAO/WHO/CODEX standard safe limit (lower than 40 ppm). Furthermore, mushrooms act as good zinc accumulators [66]; this element is highly associated with protein and carbohydrate rich foods [65]. In comparison with control mushrooms, mushrooms of S3 presented lower zinc content (reduction by 12.7%), which was in the safe limit set by the WHO (60 ppm) and reported by Gebrelibanos *et al*. [65]. The activation of some enzyme systems could be induced by trace amounts of nickel, but in high levels, this element can lead to serious toxicity [67] and cause lung cancer [68]. Nickel content increased in mushrooms produced by S2 and S3 by 5.2 and 3.1% respectively in comparison with control ones. All produced mushrooms exceeded the safe range of nickel (0.05–5 ppm) reported for plant foods [69]. S2 mushrooms were poorer in lead (reduction by 0.3%) in comparison with control ones. However, all produced mushrooms contained a high level of lead exceeding the permitted level stated by the European commission [70] (0.3 mg kg^-1^). Lead may be originated from the metallic residues left in spent coffee grounds that should be properly cleaned prior any future cultivation.

## Conclusions

A substrate combining OLPR, SCG, and wheat straw allows farmers to produce comparable mushroom yields to those obtained from the conventional straw-based substrate, offering economic advantages in regions where wheat straw is scarce. However, the tested agro-industrial residues (OLPR and SCG) may be preferably added in low proportions to wheat straw to preserve or even ameliorate some essential nutritional attributes of mushrooms, reflected by higher protein, lower fat, higher monounsaturated fatty acids, and lower sodium contents. Nevertheless, such substrates may be further tested for their effect on *P*. *ostreatus* bioactive compounds and vitamins contents.
